# ZIF-8 Derived, Nitrogen-Doped Porous Electrodes of Carbon Polyhedron Particles for High-Performance Electrosorption of Salt Ions

**DOI:** 10.1038/srep28847

**Published:** 2016-07-12

**Authors:** Nei-Ling Liu, Saikat Dutta, Rahul R. Salunkhe, Tansir Ahamad, Saad M. Alshehri, Yusuke Yamauchi, Chia-Hung Hou, Kevin C.-W. Wu

**Affiliations:** 1 Graduate School of Environmental Engineering, National Taiwan University, Taipei 10617, Taiwan; 2 Department of Chemical Engineering, National Taiwan University, Taipei 10617, Taiwan; 3 World Premier International (WPI) Research Center for Materials Nanoarchitectonics (MANA), National Institute for Materials Science (NIMS), 1-1 Namiki, Tsukuba, Ibaraki 305-0044, Japan; 4 Department of Chemistry, College of Science, King Saud University, Riyadh 11451, Saudi Arabia; 5 Australian Institute for Innovative Materials (AIIM), University of Wollongong, Squires Way, North Wollongong NSW 2500, Australia

## Abstract

Three-dimensional (3-D) ZIF-8 derived carbon polyhedrons with high nitrogen (N) content, (denoted as NC-800) are synthesized for their application as high-performance electrodes in electrosorption of salt ions. The results showed a high specific capacitance of 160.8 F·g^−1^ in 1 M NaCl at a scan rate of 5 mV·s^−1^. Notably, integration of 3-D mesopores and micropores in NC-800 achieves an excellent capacitive deionization (CDI) performance. The electrosorption of salt ions at the electrical double layer is enhanced by N-doping at the edges of a hexagonal lattice of NC-800. As evidenced, when the initial NaCl solution concentration is 1 mM, the resultant NC-800 exhibits a remarkable CDI potential with a promising salt electrosorption capacity of 8.52 mg·g^−1^.

In order to meet an overwhelmingly increasing demand for efficient anode materials for advanced electrochemical applications, nitrogen (N) doped graphitic carbon materials are found to be the desired candidate. This is because of its intrinsically superior electrical conductivity for rapid electron transport and open flexible porous structures that offering numerous active sites and short ion diffusion distances^1^,^2^,^3^. The doping of significant percentage of N into carbon matrix is considered as an ideal prospect because of the electronegativity of N (*i.e.* 3.5) and a smaller atomic diameter. In addition, the N atoms incorporated into graphitic networks facilitate the formation of stronger interactions between N-doped carbon structure and electrolyte ions (Li^+^, Na^+^) promoting their facile insertion/storage^4^,^5^,^6^, and electrosorption^7^. On the other hand, the choice of carbon matrixes is also an important task. For example, carbon materials derived via solution methods usually contain a large amount of hydroxyl, epoxy, carbonyl and carboxyl groups occupying their active sites (surfaces and edges), resulting in reduced activity. Thus, carbon materials without oxygen-bearing groups with increased N-content could offer more active sites at their edges for grafting pyridinic and pyrrolic N atoms, and such materials are highly desired for electrosorption applications. This is because, electrochemical process (sorption/storage) occurs at the edges and basal planes of carbon matrix electrode, where a pyrrolic N “hole” defect and perturbed solid electrolyte interface configuration plays a major role for the charge transfer event resulting in high surface capacitive effects^5^.

The diversity of structures and functionality of carbon nanomaterial electrodes offers tremendous opportunities for creative development for advanced electrochemical capacitive energy storage applications^8^,^9^ and recent a surge of development in this area is largely dominated by the molecular-based design to access nanoporous carbon particle of wide shapes and ranging in size from nanometers to micrometers by the thermal treatment^10^,^11^.

Metal-organic frameworks (MOFs) have been widely used for fabricating nanoporous carbon owing to their high specific surface area and controlled pore size^12^,^13^. Recently, the utilization of MOFs for producing N-doped nanoporous carbons and functional materials by virtue of dual roles such as templates and also reactive precursors has reported^14^,^15^. Despite these pioneering studies, it is still challenging to achieve a desired high level of N-doping within the hexagonal lattice and edges of MOF-derived carbon. Carbon materials with permanent nanoscale cavities and open channels are highly demanded. To meet these requirements, 3-D frameworks of zeolitic imidazolate frameworks (ZIF-8) are one of the distinguished choices to access huge N-doping into the microporous framework via direct pyrolysis. Retaining the polyhedron-like morphology in the ZIF-8 pyrolyzed product solely depends on the morphology and nature of pore networks of the parent ZIF-8. This is evident when compared pyrolysis activity of obtained ZIF-8 using low^16^ and high^17^ concentration of the structure directing agent polyvinylpyrrolidone (PVP). It is conceived that at higher concentrations of PVP higher level of N-doping and graphene-like framework with retaining shapes of parent ZIF-8 framework particles could be obtained^16^.

Capacitive deionization (CDI) is one of the promising electrochemical methods for removing salt ions from brackish water by taking advantage of electrosorption property of porous electrode in its electrical double layer region. This technology provides a great advantage in terms of drastic reduction of intrinsic energy as compared to current reverse osmosis processes^18^,^19^,^20^. An ideal carbon material with high electrosorption capacity and ion exchange rate is required for CDI electrode. Hitherto, porous carbon-based electrode materials including mesoporous carbon^21^, hierarchically porous carbon^22^ and graphenic^23^,^24^ electrodes have been mostly used and they offer maximum electrosorption capacity varied from 0.1–10 mg·g^−1^ 19. By understanding electrode structure and charge/mass transfer process it is important to develop a controllable method to construct CDI electrode material with a large capacitance, high electronic conductivity, fast responsive to ion adsorption-desorption is scientifically essential and practically useful. Doping of nitrogen would induce more defects and active site in the carbon frameworks^25^, thus the creation of a porous carbon 3-D networks with high nitrogen doping and a graphitic structure is essential because such a structure can offer large accessible pores to NaCl solution and can remarkably improve the capacitance of the electrode.

As per our knowledge, ZIF-8 derived polyhedron particles can provide a high nitrogen loading and graphitic structure together with 3-D hierarchical micro- and mesopores. Consequently, they would offer plentiful electrosorption behavior owing to the several useful features in the resulting materials, such as i) electrolyte ion reservoir in pore network, ii) moderate electrical conductivity, optimized micro-mesopore ratios, and iii) incorporation of N atoms into this polyhedron framework. In the present work, we have explored nitrogen containing ZIF-8 as the porous carbon electrode source to impart the above features into the resulting polyhedron particle electrodes.

## Results and Discussion

**Figure 1:**
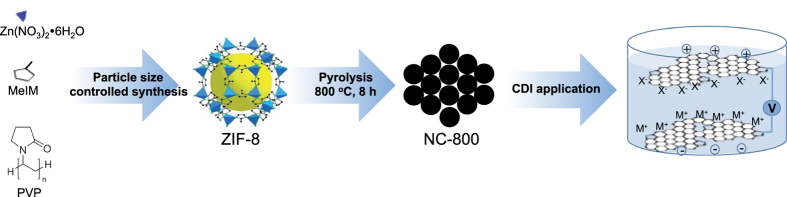
Illustration of the synthesis procedure of highly N-doped nanoporous carbon polyhedron for CDI application.

Herein, as shown in Fig. 1, we synthesize high N-doped (~15.4 Wt.%) and hierarchically porous carbon electrodes through direct pyrolysis of uniformly sized ZIF-8 via the use of an excess of polyvinylpyrrolidone (PVP) as the dispersing agent. We then demonstrate the first application of the synthesized materials in electrosorption of salt ions for desalination. Successful fabrication of CDI electrodes using hugely nitrogen doped nanoporous carbon polyhedrons enable a remarkably high salt electrosorption capacity (8.52 mg·g^−1^) for removal of Na^+^ (1.16 Å) and Cl^−^ (1.67 Å) ions present at a low level (1 mM NaCl). It is evident that the performance of electrosorption is dependent on the hierarchical porous structure of polyhedrons and nitrogen-doping in the hexagonal lattice and edges.

PVP was used as the structure directing agent and surface modifier of ZIF-8 nanoparticles, thus an organic phase synthesis (*i.e.* methanol system) of ZIF-8 from Zn(NO_3_)_2_.6H_2_O and MeIM (2-methylimidazole) with excess PVP would produce uniform polyhedron shaped ZIF-8 nanoparticles. The as-synthesized ZIF-8 polyhedrons were then heated at high temperatures (value) under N_2_ atmosphere to generate highly nitrogen doped, hierarchically porous, graphitic carbon nanoparticles (Fig. 1). TGA analysis of the heat-treated ZIF-8 (Fig. S1) indicates several weight loss steps. The first two steps of weight loss occurred at 30–230 °C and 230–490 °C, this is attributed to the removal of physisorbed water molecules and the decomposition of oxygen-containing functional groups, respectively. Further decomposition occurred around 710 °C due to the formation of graphitic framework and the release of nitrogen-containing gases. Therefore, we conclude that the synthesized ZIF-8 samples at 800 °C to fully convert from the amorphous carbon framework to graphitic framework, and the calcined sample is named as NC-800.

**Figure 2:**
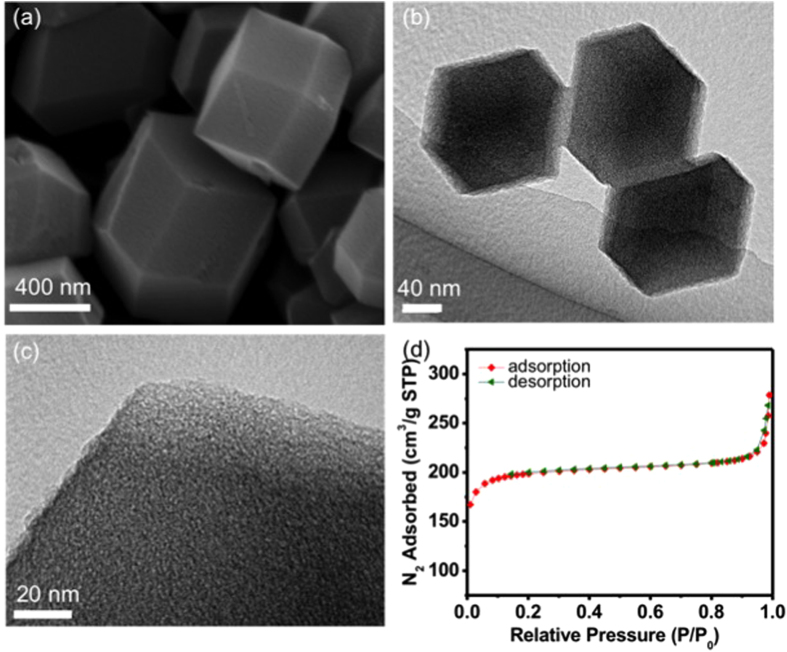
(**a**) A FE-SEM image, (**b**,**c**) TEM and HR-TEM images, and (**d**) a typical nitrogen adsorption/desorption isotherm of the ZIF-8-derived NC-800 nanoparticles.

The polyhedron morphology of the synthesized samples was observed by field emission scanning electron microscopy (FESEM). As shown in Fig. 2a, it shows that the NC-800 sample retains regular polyhedron morphology of the parent ZIF-8. The high-resolution transmission electron microscope (HRTEM) images of the NC-800 (Fig. 2b,c) reveals the porous structure in the ZIF-8 derived carbon polyhedron. As shown in Fig. S2, the images of elemental mapping by SEM microscopy confirms that a large quantity of N atoms are effectively incorporated into the framework of the NC-800 polyhedrons. High-resolution TEM images of NC-800 are as shown in the Fig. S3. The porosity of these NC-800 samples was examined with nitrogen adsorption/desorption isotherms. As shown in Fig. 2d and Table 1, the BET specific surface area and the main micropore size of the NC-800 are estimated to be 798 m^2^·g^−1^ and around 0.8 nm, respectively. Furthermore, it can be found that micropores contribute the most to this high surface area in NC-800, according to the high ratio of S_mic_/S_BET_ = 95%. The surface area, pore sizes, and the relative ratios of micropores can be varied, depending on several parameters, such as precursor concentrations, additives, aging conditions, and calcination temperatures^26^,^27^.

The carbon framework of the NC-800 was characterized with X-ray diffraction (XRD) and Raman spectra. In contrast to parent ZIF-8, the NC-800 exhibited a broad XRD peak at approximately 24°, which corresponds to the (002) peak of a graphitic carbon material (Fig. S4). In addition, the Raman spectra of the NC-800 (Fig. S5) displays the presence of G* and 2D band in addition to D and G bands which are the characteristic feature of graphene layers. The 2D-band corresponds to the disordered carbon or defective graphitic structures. The ratio of the band intensity (*i.e.* I_D_/I_G_) is calculated to be around 1.2, indicating a large amount of defects by sp^3^ carbons. These defects could be resulted from the edge modification of NC particles by N atoms.

The amount and the chemical structure of the N atoms in NC-800 were examined with elemental analysis (EA) and X-ray photoelectron (XPS) spectroscopy. The amount of nitrogen in NC-800 was determined to be 18.7 or 15.4 wt% by EA or XPS, respectively. These values are close to the value of the nitrogen content in ZIF-8 (i.e. 17.7 wt%)^13^. In addition to the nitrogen source from the imidazole group in ZIF-8, we suggest that the PVP polymer which was used as the dispersing agent would also contribute a certain amount of nitrogen.

The N1s spectrum of XPS (Fig. S6) for NC-800 can be deconvoluted to three peaks: pyridinic-N (N-6 398.4 ± 0.2), and quaternary N (N-Q, 400.7 ± 0.4). The N-6 species is the dominant N-containing functional group in the NC-800 sample (*i.e.* 82.8%), and these N-6 species serve as electrochemically active sites for enhancing the capacitive behaviors. The percentage of the N-Q species is about 17.2%, and it refers to graphitic nitrogen, which locates inside the graphitic carbon framework. The large percentage of N-Q species in NC-800 indicates an increase in the degree of graphitization of ZIF-8 framework during pyrolysis at 800 °C.

To evaluate the electrochemical performance of the synthesized NC-800 as electrodes, electrochemical impedance spectroscopy (EIS), galvanostatic charge-discharge (GC), and cyclic voltammetry (CV) measurements were performed in NaCl electrolyte solutions. To characterize the electrical conductivity, we studied the Nyquist plot of the EIS for NC-800. As shown in Fig. 2a, the Nyquist plot was constituted by two regions between Z′ (real axis) and Z″ (imaginary axis), corresponding to a semicircle at high-frequency and a straight line in the low frequency region. In the high-frequency region, the semicircle expressed the equivalent series resistance (ESR), reflecting the diffusion and transport of ions in the electrolyte^22^. Notably, the quasi-semicircle of the NC-800 with small arc size is observed, suggesting a low charge transfer resistance. In the low-frequency region, the straight line is evident for an ideal electrical double layer capacitance and faster ion diffusion behavior results from low Warburg diffusion resistance. Hence, the NC-800 electrode has a favorable accessibility of ions. This result reflects the fact that the NC-800 electrode exhibits good charge storage behavior with the electrical double layer capacitive (EDLC) mechanism.

**Figure 3:**
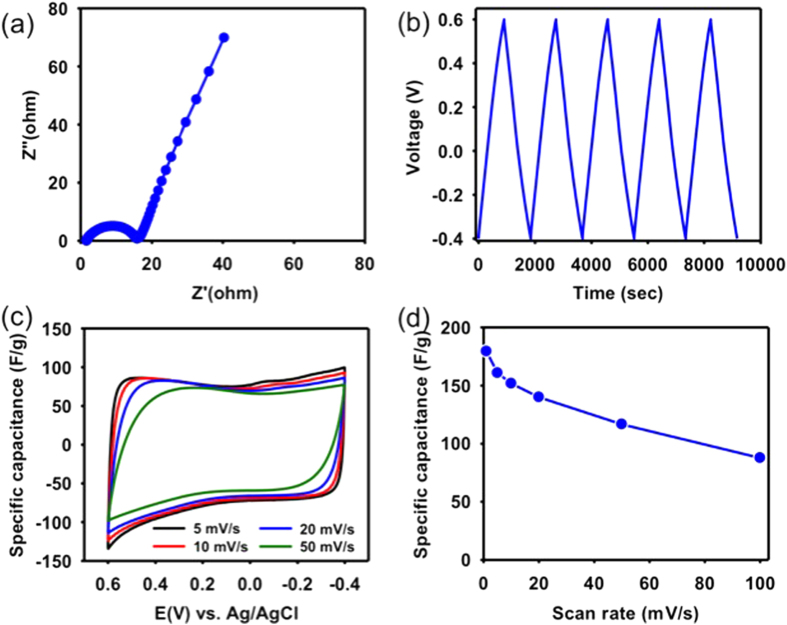
(**a**) EIS analysis of NC-800 presented as Nyquist plot, (**b**) galvanostatic charge/discharge curve of NC-800 with a current load of 0.1 A·g^−1^, (**c**) cyclic voltammograms of NC-800 at various scan rates, (**d**) specific capacitance values at various scan rates for NC-800 sample. All the experiments were carried out in a 1 M NaCl electrolyte solution.

Figure 3b shows the GC curve of NC-800 electrode at a current density of 0.1 A g^−1^ in 1 M NaCl. As observed, the curve displays a symmetric and triangular shape with a negligible potential drop (iR drop) even at such low current density, indicating to the good reversibility and non-Faradic reaction.

This confirms that the NC-800 electrode has a good electrical double layer behavior for ion storage^23^. Moreover, a comparative test of GC curves for NC-800 was carried out in 1 M and 0.01 M (Fig. S7). It can be seen that at a high concentration of 1 M NaCl, a longer charge-discharge time of NC-800 electrode was needed, corresponding to a higher capacitive charge storage feature. Further, iR drop at the tuning point of GC profile is indicative of an inner resistance of ion transport into this porous structure. As demonstrated, the iR drop in 1 M and 0.01 M NaCl were 0.01 V and 0.08 V, respectively. This implies that the mass transfer of salt ions at low NaCl concentrations such as 0.01 M, referred to as brackish water, may be restricted through the pore network. With this regard, charge transfer enhancement to capture salt ions in electrosorption process is pursed for CDI electrodes.

CV measurements of NC-800 were performed in 1, 0.1, 0.01, 0.001 M NaCl at various scan rates in a range of 1 mV s^−1^ to 100 mV s^−1^ (as shown in the Fig. S8 and Table S1). As seen, from the CV curves, NC-800 electrodes have an apparently rectangular shape at high-concentrations of NaCl solution with a slow scan rate such as 1 mV·s^−1^ and 5 mV·s^−1^. Meanwhile, the current response shows no evidence for redox peaks within the potential range of −0.4 to +0.6 V, corresponding to the formation of electrical double layer. Moreover, Fig. 3c shows the CV curves of NC-800 electrode in 1 M NaCl at different scan ratesranging from 5 to 50 mV s^−1^. It should be noted that with increasing the scan rate, ions barely have time to be transported from bulk solution to the 3-D pore network to fully develop the double layer. As seen, the CV curves of NC-800 were slightly distorted, but still presented nearly quasi-rectangular shape. As calculated by equation (1) (refer to Supporting Information), Fig. 3d shows the specific capacitances of NC-800 in 1 M NaCl solution as a function of scan rate. Clearly, the specific capacitance decreases with increasing the scan rate. The scan rate dependence is a typical capacitive behavior for the microporous structure of ZIF-8 derived carbon material^28^. It should be emphasized that the NC-800 exhibits superiority in the specific capacitance of capacitive charge storage over other ZIF-8 derived carbon materials calcined at lower temperatures in all the electrochemical measurements. Fig. S9 displays the EIS profile, GC, and CV curves of the NC-700 (*i.e.* ZIF-8 derived carbon calcined at 700 °C). For example, with an increase of scan rate from 5 to 50 mV s^−1^, the specific capacitance of NC-700 considerably decreased from 135.5 to 18.6 F g^−1^, respectively. In the meantime, the NC-800 remained high specific capacitances of 160.8 F g^−1^ and 116.7 F g^−1^ at 5 mV s^−1^ and 50 mV s^−1^, respectively. Notably, the NC-800 presents better capacitive properties and has a less scan rate dependence on the specific capacitance as compared to the NC-700, reflecting a better rate capability for electrosorption of ions. In brief, the NC-800 associated with good electrochemical performance can be considered as a favorable candidate for CDI, and thereby, it was selected for the following CDI experiment.

**Figure 4:**
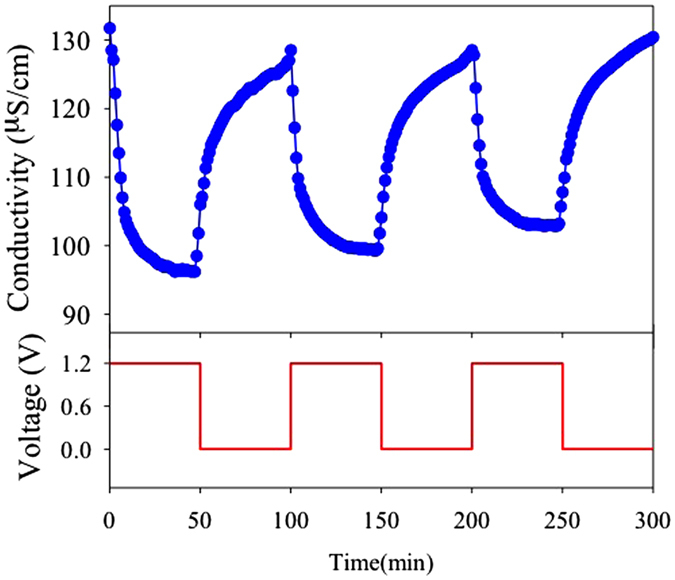
Electrosorption-regeneration cycles of Na^+^ ions from 1 mM NaCl solution using ZIF-8-derived NC800 electrode with an applied voltage of 1.2 V.

CDI performance of the NC-800 electrode was investigated by batch-mode experiment at a applied voltage of 1.2 V. The initial concentration of the NaCl solution was 1 mM (~58 mg L^−1^) with an initial conductivity of about 131 μS cm^−1^. Figure 4 shows the electrosorption and regeneration cycles of the NC-800 by repeating the charge-discharge process three times. When the voltage was applied on a pair of working electrodes, a dramatic decrease of the solution conductivity was observed at the early stage. It suggests the fast charge transfer of salt ions from the bulk solution into the charged pores. Then, the conductivity gradually decreased to 96 μS cm^−1^ at pseudo-equilibrium in the first cycle. After the voltage was removed, the ions were released back to the bulk solution, and thus, the solution conductivity returned to about the initial value. This finding indicates that NaCl removal is mainly ascribed to an electrostatic interaction of electrical double layer formation. The same pattern of electrosorption-desorption curves, corresponding to the good regeneration performance, further demonstrates that the NC-800 electrode has good electrochemical stability and reversibility in the electrosorption process. Furthermore, the electrosorption capacity of the NC-800 was calculated (using the equation 2 in supporting information) to be 8.52 mg g^−1^.

For comparison, we list all salt electrosorption capacities of other porous carbon-based materials for CDI (Table S2). As demonstrated, the conventional CDI electrodes have electrosorption capacity in a range from 2 to 7 mg g^−1^ for desalting NaCl solution at relatively low concentrations. We have previously used cellulose fibers as the template to synthesize hierarchically porous carbon (HPC) for CDI application, and an electrosorption capacity as high as 7.75 mg g^−1^ was achieved^22^. Here, our NC-800 electrode exhibits even higher electrosorption capacity (*i.e.* 8.52 mg g^−1^). We believe that the superior electrosorption capacity for salt ions was owing to the high specific surface area of NC-800 associated with the edge-sharing of framework nitrogens (creating additional defect sites). In addition, the small particle size of NC-800 with interconnected micropores can ensure the fast charge transfer for capacitive ion storage.

In conclusions, we synthesize high surface-nitrogen doped carbon polyhedron particles through the pyrolysis of ZIF-8 nanoparticles. ZIF-8-derived carbon polyhedron particles performed excellently in electrosorption-based salt ion capture from saline water at a low applied voltage. Experimental evidence from the structural studies of these materials and its correlation with the CDI experimental results on salt-ion capture supports the hypothesis of multiple advantages of ZIF-8 derived carbon nanoparticles as electrodes. It is understood that, edge-sharing of framework nitrogen and microporous framework of ZIF-8-derived carbon particles based electrodes plays a major role in enhancing the electrosorption capacity of the electrode over a range of a structurally diverse set of electrodes. We expect that the ZIF-8 derived NCs material could also be considered as high-performance electrodes for other energy or environmental issues.

## Methods

### Chemicals

Zinc nitrate hexahydrate (≥99%), 2-methylimidazole (99%), polyvinylpyrrolidone (PVP, K30, MW 40,000) were purchased from Sigma-Aldrich. All chemicals were used without any further purification.

### Preparation of the surfactant-controlled zeolitic imidazolate framework, ZIF-8

During the typical synthesis procedure, a methanolic solution (50 ml) of zinc nitrate (Zn(NO_3_)_2_·6H_2_O, 2.4 g) was added dropwise to a methanolic solution (50 ml) of 2-methylimidazole (2.1 g) and polyvinylpyrrolidone (K-30, 6.0 g) using a syringe while stirring at room temperature. The entire reaction process was performed at room temperature with agitated stirring for 20 min. The reaction was aged at room temperature without any interruption for 10 h. The resulting white precipitate was centrifuged and washed several times with methanol before drying in an oven at 60 °C.

### Preparation of the N-doped graphitic carbon polyhedron particles (NC)

The N-doped graphitic carbon particles were synthesized by direct carbonization of the as-prepared ZIF-8 under a flow of nitrogen gas at various temperatures. Typically, the ground ZIF-8 was homogeneous.

## Additional Information

**How to cite this article**: Liu, N.-L. *et al.* ZIF-8 Derived, Nitrogen-Doped Porous Electrodes of Carbon Polyhedron Particles for High-Performance Electrosorption of Salt Ions. *Sci. Rep.* **6**, 28847; doi: 10.1038/srep28847 (2016).

## Supplementary Material

Supplementary Information

## Figures and Tables

**Table 1:** Porous characteristics of the NC-800 sample.

Sample	Specific surface area (m^2^g^−1^) (***S***_BET_)	Micropore diameter (nm)^[b]^	Total pore volume (cm^3^g^−1^)^[c]^	Micropore volume (cm^3^g^−1^)^[c]^	Micropore surface area (m^2^g^−1^) (***S***_mic_)^[c]^	***S***_mic_ */**S***_BET_ (%)	I_D_/I_G_ (Raman spectral intensity)^[d]^
NC800	798	0.8	0.50	0.40	760	95	1.2

^[a]^BET method, ^[b]^NLDFT method, ^[c]^t-plot method, ^[d]^Peak intensity of D and G bands in Raman spectra.
