# Supplementary material for: ZIF-8 Derived, Nitrogen-Doped Porous Electrodes of Carbon Polyhedron Particles for High-Performance Electrosorption of Salt Ions
Source: Sci Rep. 2016 Jul 12;6:28847. doi: 10.1038/srep28847 (PMC4941406; doi:10.1038/srep28847)
Supplement: Supplementary Information [file srep28847-s1.pdf]

ZIF-8 Derived, Nitrogen-Doped Porous Electrodes of Carbon Polyhedron Particles: Framework Nitrogen and Hierarchical Porosity for High-Performance Electrosorption of Salt Ions

Nei-Ling Liu,[1] Saikat Dutta,[2] Rahul R. Salunkhe.,[3] Tansir Ahamad,[4] Saad M. Alshehri,[4] Yusuke Yamauchi,[3] Chia-Hung Hou,[1]* and Kevin C.-W. Wu[2]*

1. Graduate School of Environmental Engineering, National Taiwan University, Taipei 10617, Taiwan.

2. Department of Chemical Engineering, National Taiwan University, Taipei 10617, Taiwan.

3. World Premier International (WPI) Research Center for Materials Nanoarchitectonics (MANA), National Institute for Materials Science (NIMS), 1-1 Namiki, Tsukuba, Ibaraki 305-0044, Japan.

4. Department of Chemistry, College of Science, King Saud University, Riyadh 11451, Saudi Arabia.

Equal contribution for the first two authors.

E-mail addresses of corresponding authors: chiahunghou@ntu.edu.tw (C.H. Hou) and kevinwu@ntu.edu.tw (K.C.W. Wu)

*Supporting information for this article is given via a link at the end of the document.

EXPERIMENT SECTION:

1. Electrochemical Experiments:

A CH instruments (CHI 627D) electrochemical workstation was used to perform the electrochemical experiments, including electrochemical impedance spectra (EIS), galvanostatic charge/discharge (GC) and cyclic voltammetry (CV) measurements. All the electrochemical measurements were carried out using a three-electrode cell system in a NaCl solution An Ag/AgCl (sat.) (CHI 111) act as the reference electrode. Electrochemical impedance spectra (EIS) measurement was performed to determine

the electrical conductivity of the carbon electrode. The data was collected in the frequency range from 10 mHz to 100 kHz with 5 mM a amplitude. The GC test was used to measure the reversibility and inner resistance (iR drop) of the carbon electrode. Voltage profiles were obtained at a current density of 100 m $A \cdot g^{-1}$ in the potential range of −0.4 to 0.6 V. The CV measurement was executed to evaluate the capacitive performance of the carbon electrode. CV for assessing the EDL capacitance were measured in a potential window −0.4 to 0.6 V at various scan rate, ranging from 5 to 1000 $mV \cdot s^{-1}$. The specific capacitance derived from the CV curves can be estimated according to the following equation:

$$C = \frac{\int_{V_a}^{V_c} I \, dV}{mv(V_c - V_a)} \quad (1)$$

where C is the specific capacitance, v is the scan rate, m is the mass of the carbon material, and I is the current density. V_c and V_a represent the high and low potential limits of the cyclic voltammetry tests.

2. CDI Application of ZIF-8-derived NC-800:

The CDI electrodes were prepared by mixing a slurry of 90 wt% porous carbon powder and 10 wt% of polyvinylidene fluoride (PVDF, M.W. = 534,000, Sigma-Aldrich) binder in N, N-Dimethylacetamide (DMAc, 99%, Alfa Aesar) solution, followed by stirring for 12 h to ensure homogeneity. The slurry of the mixture was coated onto a titanium plate, dried in a 120 °C oven for 2h and in a 80 °C vacuum oven for 2h to remove organic solvents.

The CDI experiments of NCs were conducted in a batch-mode recycling system, in which the NaCl solution was continuously circulated through the CDI unit cell using a peristaltic pump (EYELA MP-1000) at a flow rate of 5 $mL \cdot min^{-1}$. The CDI unit cell consisted of a couple of carbon electrodes and a pair of titanium plates as the current collectors, which were separated by a spacer by the distance of 2 mm for solution flow. Prior to each experiment, the CDI cell was flushing by 18-ΩM deionized water until the solution conductivity decreased to value near zero. The electrical voltage of 0.8, 1.0 or

1.2 V was applied to the two carbon electrodes using a CHI 627D potentiostat. The regeneration was carried out by discharging the cell at 0 V. The change in solution conductivity was also continuously monitored at the outlet of the CDI cell by an online conductivity meter (SC-2300, Suntex). The concentration of the NaCl electrolyte was further determined by the linear relationship between the NaCl concentration and the conductivity in solution. The electrosorption capacity (*Q*) is calculated as the following equation:

$$Q = \frac{(C_0 - C_e)\cdot V_{NaCl}}{m} \qquad (2)$$

where C_0 and C_e are the initial and equilibrium concentrations, respectively, and V_{NaCl} is the solution volume.

3. Characterization:

SEM images of the samples were recorded by using a scanning electron microscope (Nova Nano SEM) operating at an acceleration voltage of 5.00 kV. High-resolution transmission electron microscopy (HR-TEM) images were obtained by using a JEOL JEM-2100F microscope operated at an accelerating voltage of 200 kV. Thermo gravimetric analyze (TGA) of samples were conducted by using a SDT Q600 thermo gravimetric analyzer in N_2 from room temperature to 900 °C at a heating rate of 10 °C min^{-1}. X-ray photoelectron spectroscopy was collected on a Thermo Scientific spectrometer of type “Sigma Probe” and XPS spectra of the samples were further deconvoluted into several narrow-scan spectra of the C_{1s}, O_{1s} and N_{1s} by using the software Avantage Data System (Version 3.95). XRD patterns were performed on a D8 ADVANCE (Germany) using Cu-*K*α (0.15406 nm) radiant ion. The porous properties were analyzed using nitrogen adsorption/desorption isotherms on a Micromeritics ASAP 2010 instrument. The specific surface area and pore size were calculated using the Brunauer–Emmet–Teller (BET) and Non-Linear Density Functional Theory (NLDFT) methods, respectively. The resulting pore size distribution (PSD) curves defined as dV/d(logD), where V is the adsorbed volume and D is the pore width. Micropore data of the NC-800 were obtained in Micromeritics ASAP 2010 instrument and the data were analyzed by using Original Density Functional Theory Model by the method of Non-

negative Regularization. X-ray photoelectron spectroscopy (XPS) was measured at room temperature using a Thermo Scientific instrument with Theta Probe with an Al Kα X-ray source of 1486.6 eV (AlKαline) and take-off angle (TOA) was set to 53°, pass energy: 20 eV; energy step 0.05 eV; scan no.: 30. The region of survey spectra is 0 to 1400 eV and the region of high-resolution N1s spectra is 392 to 410 eV. The percentage of N was calculated from the XPS survey spectrum by using N1s peak. All the binding energies were calibrated via referencing to C1s binding energy (285.0 eV).

RESULTS AND DISCUSSION SECTION:

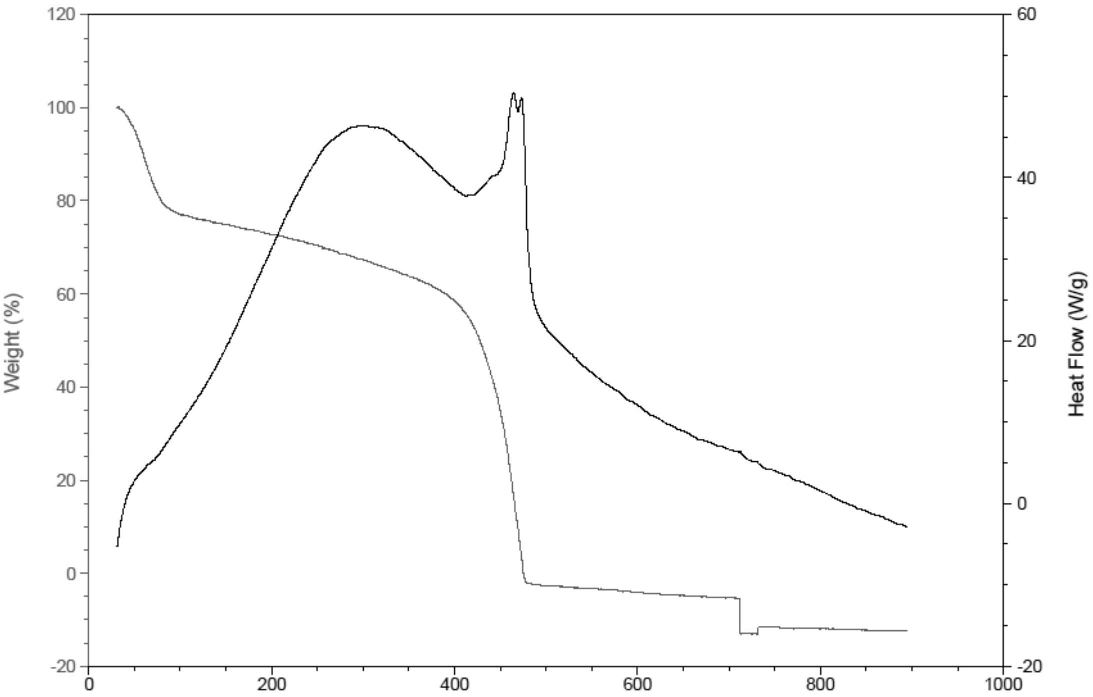

Figure S1. DSC-TGA of the synthesized ZIF-8 nanoparticles.

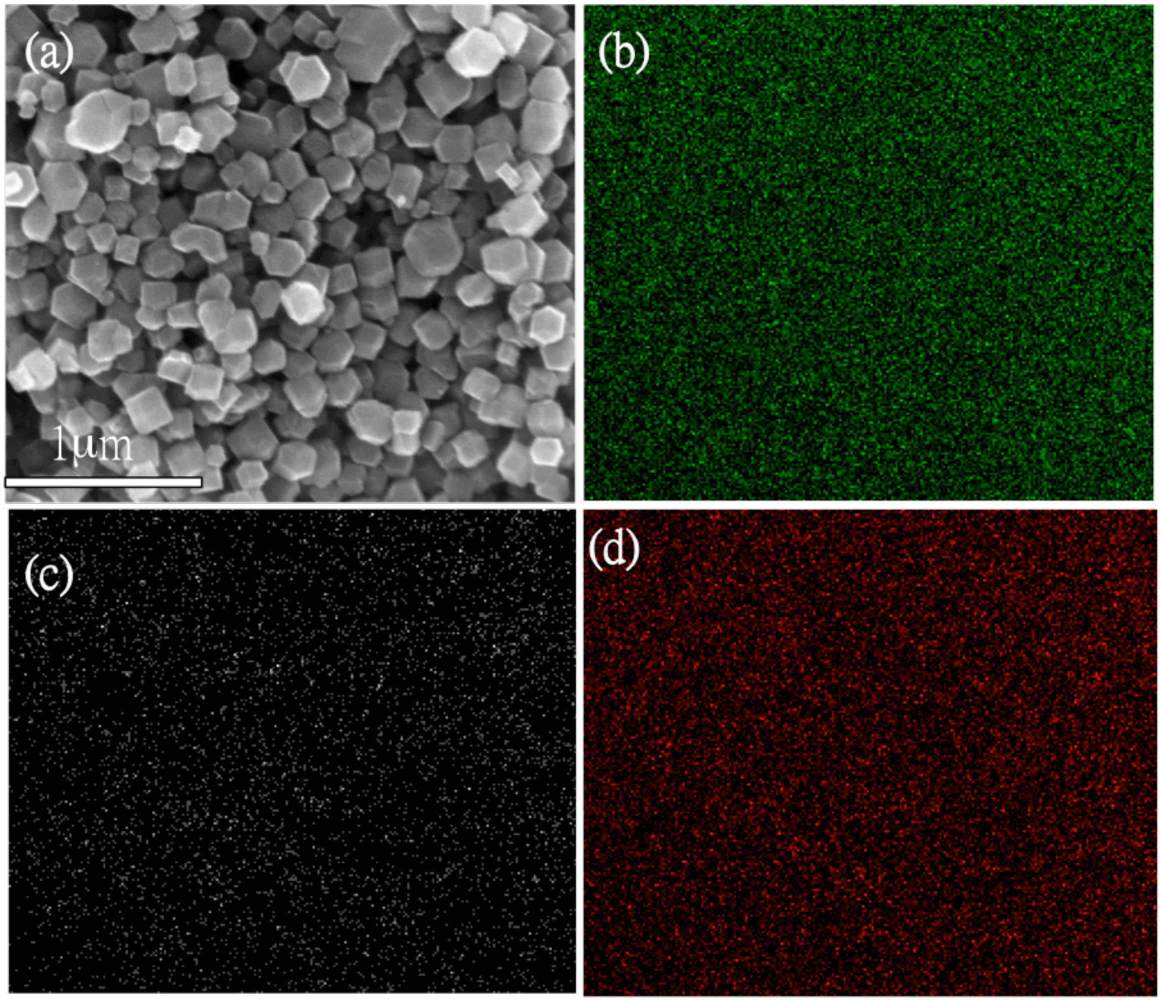

Figure S2. (a) A SEM image for typical NC-800 sample, and its elemental mapping: (b) carbon, (c) nitrogen and (d) oxygen element.

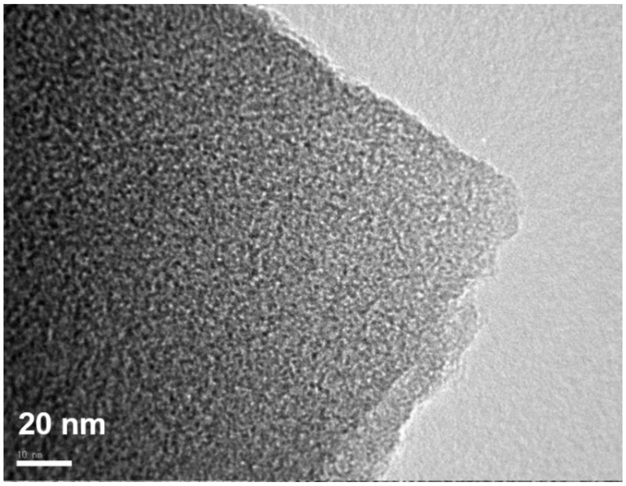

Figure S3. A HR-TEM image of ZIF-8 derived NC-800 sample.

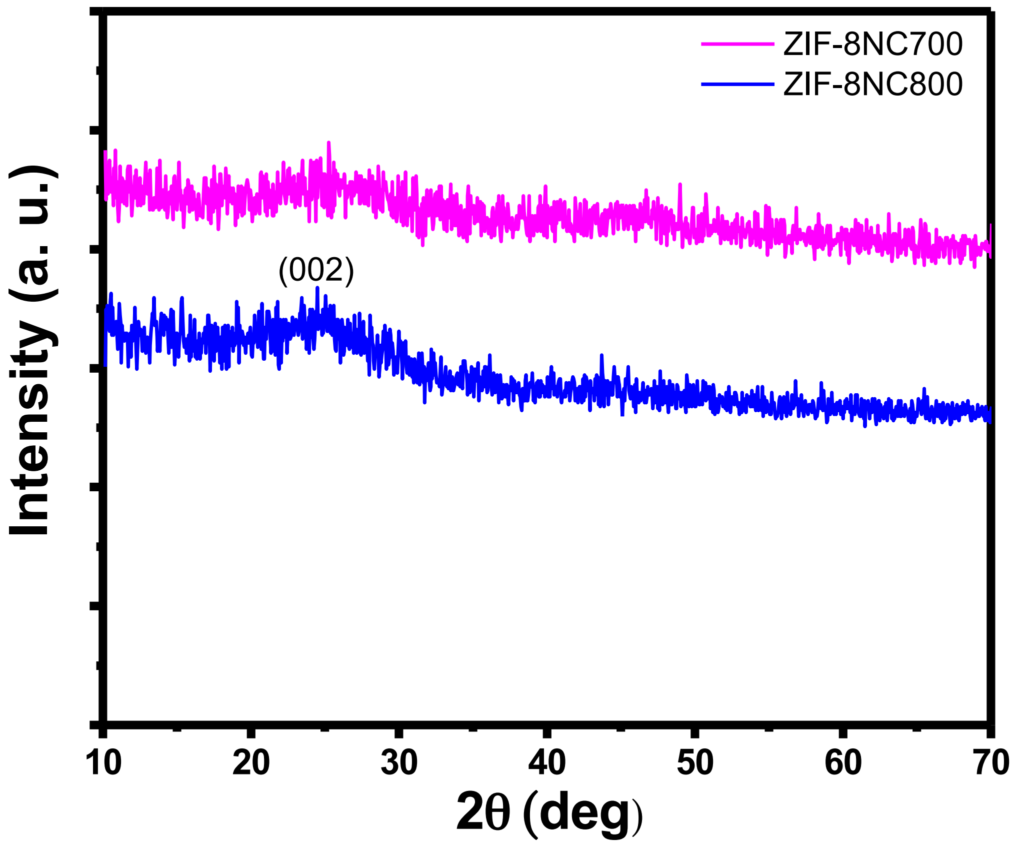

Figure S4. XRD patterns of ZIF-8 derived samples (NC-700 and NC-800).

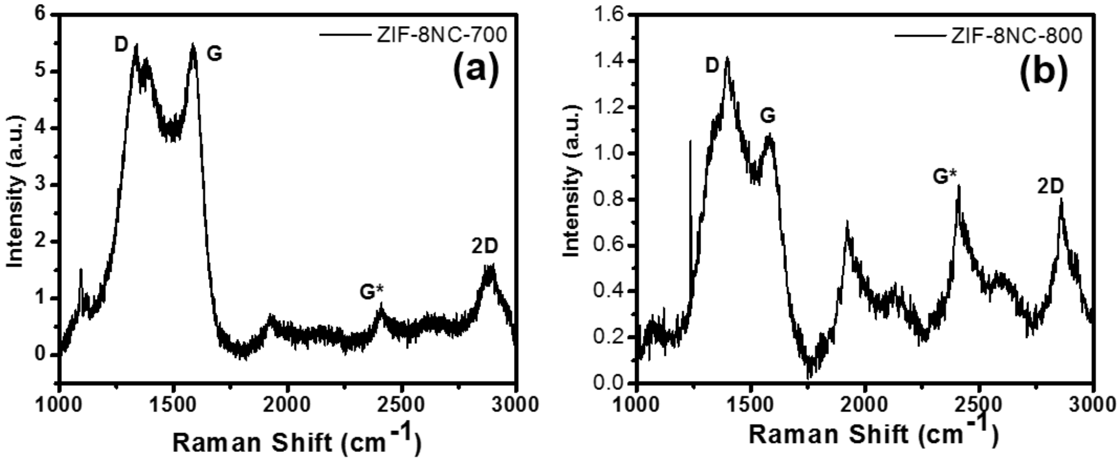

Figure S5. Raman spectra of NC samples ((a) NC-700 and (b) NC-800) exhibiting evolution of D and G band at an increasing I_D/I_G with increased pyrolysis temperature of ZIF-8 and also showing evolution of secondary bands G* (2450 cm^{-1}) and 2D (2700 cm^{-1}) characteristic of presence of graphene layers.

Table S1. Chemical composition of NC-800 measured by elemental analysis (EA), XPS, and EDX spectroscopy.

Material		C (wt%)	N (wt%)	H (wt%)
NC-800	EA	48.37	18.69	3.17
	XPS	64.80	15.40	19.80
	EDX	59.72	34.87	NA

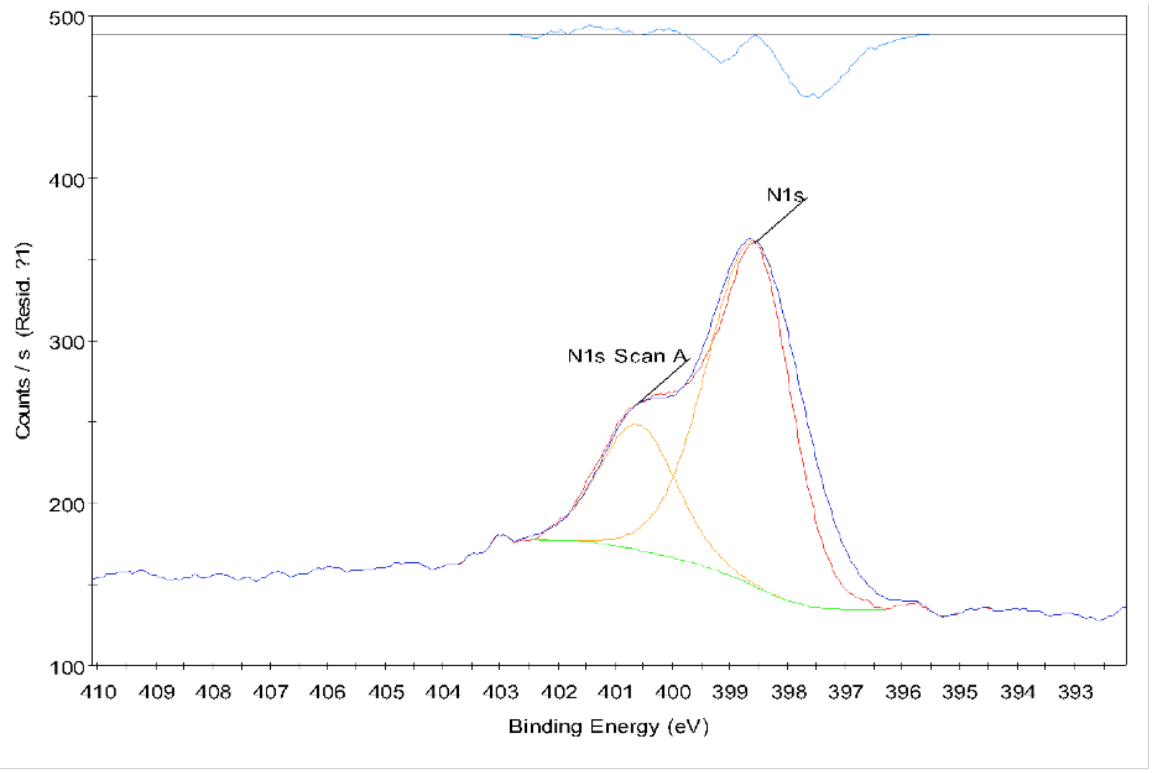

Figure S6. The corresponding deconvoluted spectra high-resolution N1S XPS spectra of NC-800.

Table S2. Distribution (%) of surface N-functional groups by fitting the N1s core level XPS spectrum for the NC-800.

	% of total N1s		
Samples	N-6 (pyridinic N)	N-5 (pyrrolic/pyridonic N)	Quaternary N
BE (eV)	398.4± 0.2	399.8 ± 0.2	400.7 ± 0.4
NC-800		398.56 (82.80%)	400.6 (17.16%)

Table S3. Distribution (%) of surface C-functional groups by fitting the C1s core level XPS spectrum for the NC-800.

Samples	sp^2 graphitic C	C-OH
BE (eV)	283-284	285
NC-800	284.69 (86.57%)	286.25 (13.86%)

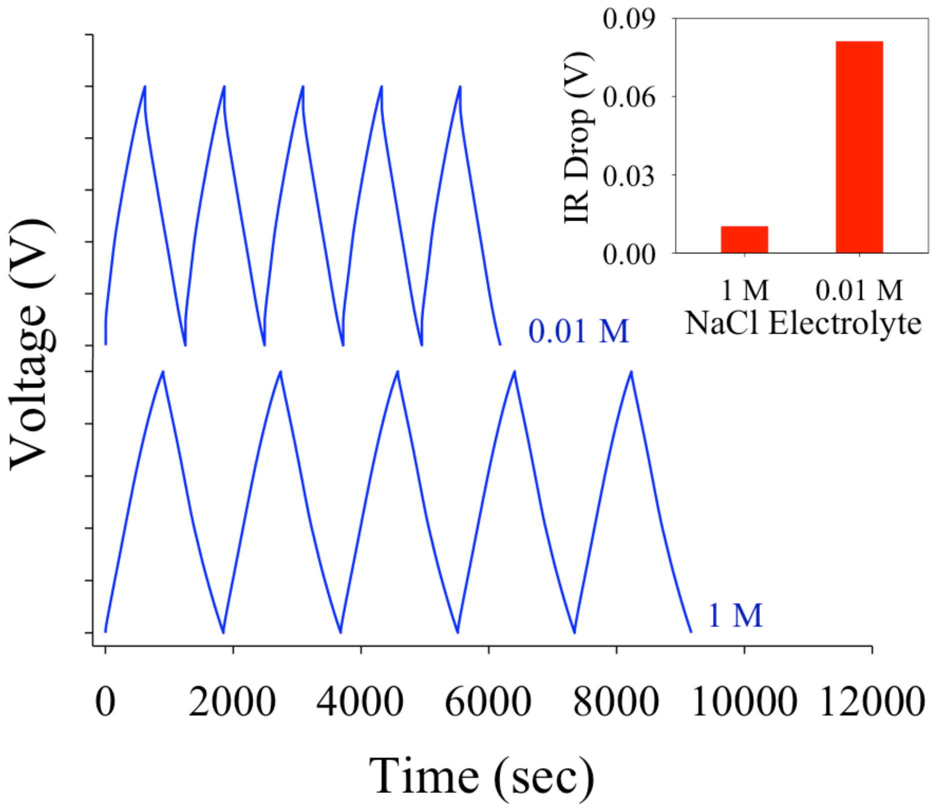

Figure S7. Galvanostatic charge/discharge curve of NC-800 carbon electrode in 1 M NaCl solutions with scan window from -0.4 V to 0.6 V and a current load of 0.1 A/g.

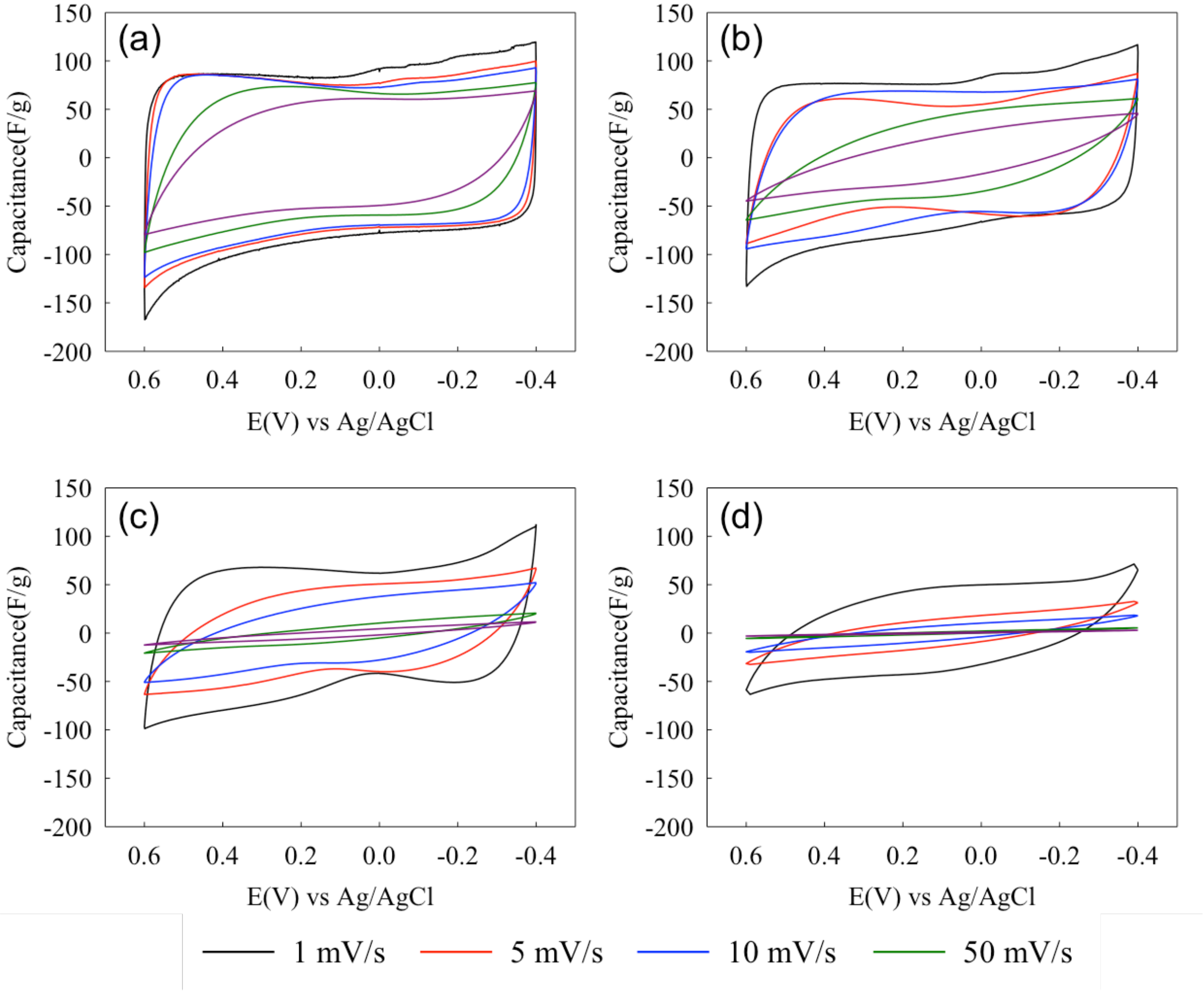

Figure S8. CV curves of NC-800 at various NaCl concentrations (a) 1 M, (b) 0.1 M, (c) 0.01 M and (d) 0.001 M at various applied voltages (1, 5, 10 and 50 mV/s).

Table S4. Specific capacitance of NC-800 at the scan rate of 1-200 mV/s in 1-0.001 M NaCl (cyclic voltammetry experiment).

	CV Capacitance at varied scan rates					
ZIF-8NC-800	100 mV/s	50 mV/s	20 mV/s	10 mV/s	5 mV/s	1 mV/s
1MNaCl	87.76	116.67	140.02	151.74	160.77	179.41
0.1MNaCl	31.57	59.05	96.66	119.44	137.58	155.49
0.01MNaCl	4.66	10.92	26.22	46.83	71.76	118.61
0.001MNaCl	0.38	0.76	9.28	19.10	36.64	106.06

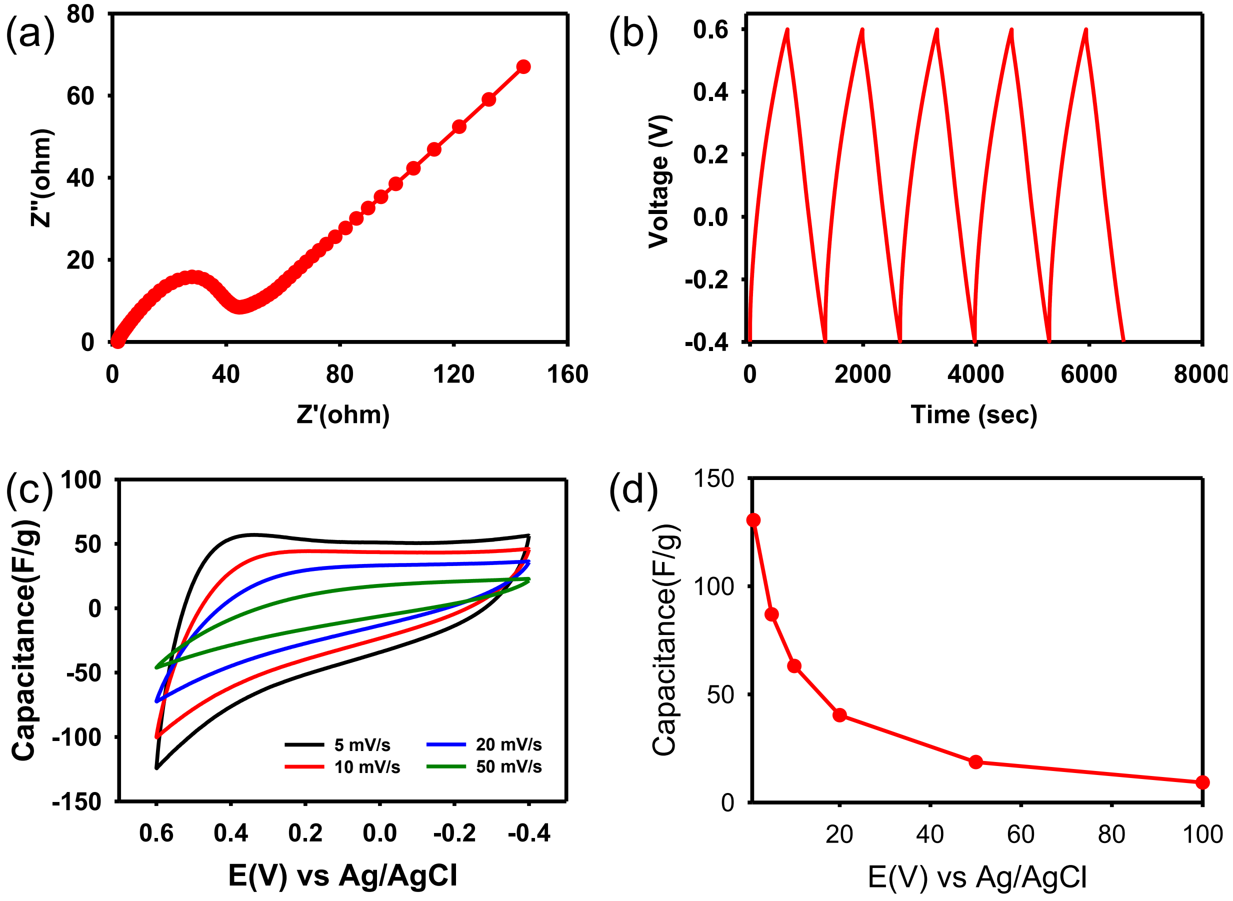

Figure S9. (a) EIS analysis of NC-700 presented as Nyquist plot, (b) Galvanostatic charge/discharge curve of NC-700 with a current load of 0.1 A/g, (c) Cyclic voltammograms of NC-700 at various scan rates, Specific capacitances of NC-800 vs scan rate. All experiments were carried out in a 1 M NaCl electrolyte solution.

Table S5. Comparison of NaCl electrosorption capacity of various porous carbon electrodes by CDI method for brackish water.

Carbon Electrode	Surface Area ($m^2 \cdot g^{-1}$)	Pore volume (cm^3/g)	Initial NaCl concentration (mg/L)	Applied voltage (V)	Electrosorption capacity ($mg \cdot g^{-1}$)	References
3DGHPC	384.4	0.73	~25	1.2	6.18	[1] Wen. et al., 2013
3DG	250.3	0.49	~25	1.2	4.41	[1] Wen et al., 2013
3DHPC	1036.8	2.62	~30	2.0	2.16	[2] Wen et al., 2012
3DMGA	339	-	~50	1.2<br>1.6<br>2.0	1.97<br>3.90<br>5.39	[3] Wang et al., 2013
OMC	844	0.90	~25	1.2	0.68	[4] Zou et al., 2008
AC	968	0.59	~25	1.2	0.25	[4] Zou et al., 2008
Graphene	488	51.01	~25	2.0	1.30	[5] Li et al., 2010
Hierarchal ACF	428	0.138	~90	1.6	1.99	[6] Wang et al., 2012
Hierarchal OMC	410	-	~4000	1.2	14	[7] Mayes, et al., 2010
HPC-800	2535	1.50	~30	1.2	3.24	[8] Dutta et. al., 2016
NC-800	778.0	0.502	~580	1.2	7.75	This study

Reference:

[1] X. Wen, D. Zhang, T. Yan, J. Zhanga, L. Shi, *J. Mater. Chem. A*, **2013**, *1*, 12334-12344.

[2] X. Wen, D. Zhang, L. Shi, H. Wang, J. Zhang, *J. Mater. Chem.* **2012**, *22*, 23835.

[3] H. Wang, D. Zhang, T. Yan, X. Wen, J. Zhang, L. Shi, Q. Zhong, *J. Mater. Chem. A*, **2013**, *1*, 11778-11789.

[4] L. Zou, L. Lib, H. Song, G. Morrisa, *Water Res*. **2008**, *42*, 2340-2348.

[5] H. Li, L. Zou, L. Pam, Z, Sun, *Environ. Sci. Technol.*, **2010**, 44, 8692-8697.

[6] G. Wang, Q. Dong, Z. Ling, C. Pan, C. Yu, J. Qiu, 2012, *J. Mater. Chem*. **2012**, 22, 21819.

[7] R.T. Mayes, C. Tsouris, J. O. Kiggans Jr., S. M. Mahurin, D.W. DePaoli, S. Dai, *J. Mater. Chem*. **2010**, 20, 8674-8678.

[8] S. Dutta, S.-Y. Huang, C. Chen, H. E. Chen, Z. A. Alothman, Y. Yamauchi, C.-H. Hou, K. C. W. Wu, *ACS Sustainable Chem. Eng*. **2016**, *4*, 1885-1893.
